# Combination of high-flow nasal oxygen and ketamine/dexmedetomidine sedation for diagnostic catheterization in a child with pulmonary arterial hypertension: a case report

**DOI:** 10.1186/s40981-024-00699-z

**Published:** 2024-02-22

**Authors:** Kaoru Tsuboi, Misuzu Asai, Toshiki Nakamura, Jun Ninagawa, Hiroshi Ono, Shugo Kasuya

**Affiliations:** 1 Department of Critical Care and Anesthesia, National Center for Child Health and Development, 2-10-1 Okura, Setagaya-ku, Tokyo, Japan; 2 Department of Anesthesiology and Critical Care Medicine, Yokohama City University Hospital, 3-9 Fukuura, Kanazawa-Ku, Yokohama, Japan; 3 Department of Cardiology, National Center for Child Health and Development, 2-10-1 Okura, Setagaya-ku, Tokyo, Japan

**Keywords:** Pulmonary arterial hypertension, Cardiac catheterization, Anesthesia, Pediatrics

## Abstract

Pulmonary hypertension is associated with significant risk of perioperative life-threatening events. We present a case of a 12-year-old child with severe pulmonary arterial hypertension who successfully underwent diagnostic cardiac catheterization under ketamine and dexmedetomidine sedation with the support of high-flow nasal oxygen. Ketamine and dexmedetomidine are reported to have minimal effect on pulmonary vasculature in children with pulmonary hypertension and can be safely used in this population along with its lack of respiratory depression. Positive pressure generated by high-flow nasal oxygen improves upper airway patency, prevents micro-atelectasis, and is shown to improve the effectiveness of ventilation and oxygenation in patients under sedation breathing spontaneously. The presented strategy may contribute to enhancing the safety and effectiveness of procedural sedation for children with life-threatening pulmonary hypertension.

## Background

Anesthetizing children with pulmonary hypertension (PH) is associated with a significant risk of perioperative morbidity and mortality. We present a case of a 12-year-old child with severe pulmonary arterial hypertension (PAH) who successfully underwent diagnostic cardiac catheterization under sedation with the support of high-flow nasal oxygen (HFNO).

## Case presentation

A 12-year-old, 27-kg female patient with a diagnosis of idiopathic PAH was scheduled for cardiac catheterization. She was born at full term with no previous medical history. At the age of eight, she presented with exertional dyspnea, severely limited exercise tolerance, and loss of consciousness that progressed over several months. Idiopathic PAH was diagnosed. Cardiac catheterization performed under general anesthesia prior to therapeutic intervention revealed a mean pulmonary arterial pressure (PAP) of 40 mmHg and a pulmonary vascular resistance (PVR) of 11.64 Wood unit m^2^. During this procedure, she went into refractory hypotension due to excessive decrease in systemic vascular resistance (SVR) and required cardiopulmonary resuscitation, but was successfully resuscitated without complications. Oral tadalafil, masitentan, celexipag, and nocturnal home oxygen therapy were initiated, and the patient was followed up on outpatient visits without further aggravation of symptoms. Diagnostic cardiac catheterization was scheduled to assess her current cardiovascular status as well as her response to pulmonary vasodilator therapy. Echocardiography on admission showed preserved right ventricular function and a tricuspid regurgitation pressure gradient of 65 mmHg.

We planned to use ketamine in combination with dexmedetomidine under spontaneous ventilation with the support of HFNO. An inhaled nitric oxide (iNO) machine was put on standby. Midazolam 0.05 mg/kg, ketamine 1 mg/kg, and atropine sulfate 0.01 mg/kg IV were administered, followed by a loading dose of dexmedetomidine 1 mcg/kg IV over 10 min. HFNO was administered at 35 L/min with fraction of inspired oxygen (F_I_O_2_) of 0.21 under spontaneous breathing. Catheter access in the femoral vessels was preceded by infiltration of 0.5% lidocaine and two additional doses of ketamine 1 mg/kg as body movements were observed at the timing of lidocaine injection. The level of sedation was maintained with a value of BIS around 60 under dexmedetomidine infusion at a rate of 1 to 1.5 mcg/kg/h, and the patient remained hemodynamically stable without respiratory depression throughout the procedure. Hemodynamic data and arterial blood gas analysis results are presented in Table 1. An oxygen challenge test demonstrated negative vasoreactivity. Acute vasoreactivity testing with iNO was not performed. The duration of the procedure was 40 min. Upon completion, dexmedetomidine was discontinued and HFNO was terminated. The patient spent an uneventful night in the pediatric intensive care unit and was discharged to the general ward the following morning.

**Table 1:** Hemodynamic data and arterial blood gas analysis results

	Condition 1	Condition 2
	(F_I_O_2_ 0.21, 35 L/min)	(F_I_O_2_ 1.0, 35 L/min)
**Hemodynamic data**		
SVC saturation (%)	84.5	ー
IVC saturation (%)	84.5	ー
MPA saturation (%)	78.6	90.7
Ao saturation (%)	97.3	99.9
Ao pressure (systolic/diastolic/mean) (mmHg)	104/64/80	115/69/87
MPA pressure (systolic/diastolic/mean) (mmHg)	59/28/44	53/23/37
RPAWP (mmHg)	9	10
LPAWP (mmHg)	9	ー
Rp (Wood unit m^2^)	8.42	6.9
Rs (Wood unit m^2^)	19.84	ー
Rp/Rs	0.42	ー
**Blood gas analysis**		
pH	7.378	7.377
PaO_2_ (mmHg)	99.7	498
PaCO_2_ (mmHg)	41.2	41.5
HCO_3_ (mEq/L)	23.7	23.8

Condition 2 shows oxygen challenge test. *Ao* Aorta, *IVC* Inferior vena cava, *LPAWP* Left pulmonary arterial wedge pressure, *MPA* Mean pulmonary artery, *Rp* Pulmonary arterial resistance, *RPAWP* Right pulmonary arterial wedge pressure, *Rs* Systemic arterial resistance, *SVC* Superior vena cava

## Discussion

PH remains a life-threatening condition despite current advances in therapeutic management. The risk of perioperative cardiac arrest and death is reported to be 20-fold higher in these children compared to all children undergoing anesthesia and sedation [1]. The goal of anesthetic management is to provide adequate anesthesia and analgesia for the procedure while ensuring optimal ventilation and avoiding right ventricular failure, increased PVR, low SVR, and coronary ischemia.

In this case, we sedated the child with ketamine and dexmedetomidine under spontaneous ventilation as the planned procedure was minimally invasive and was deemed to be tolerable. Ketamine is reported to have little to no pulmonary vascular effects and minimal negative effect on systemic vascular resistance, and its properties of combined anesthesia and analgesia with little impact on respiratory drive make it an attractive choice in children with PH [1–3]. Dexmedetomidine also has been shown to have minimal effect on pulmonary vasculature in children with PH and can be safely used in this population along with its lack of respiratory depression [4]. In this case, atropine sulfate was administered to prevent excessive salivation that may accompany ketamine administration although this may not have been necessary as salivation is likely offset by dexmedetomidine’s effect on the salivary glands causing xerostomia [5]. A successful case of a 12-year-old male patient with severe PAH undergoing diagnostic catheterization under ketamine and dexmedetomidine sedation has been previously reported [6].

Nevertheless, the risk for hypoventilation, atelectasis, and hypercarbia cannot be eliminated when patients undergoing sedation are breathing spontaneously through a natural airway. In order to combat these risks, we administered HFNO. HFNO is an oxygen supply system capable of delivering 100% humidified and heated oxygen at a flow rate of up to 60 L/min. Basic components include a flow generator, an air-oxygen blender that allows F_I_O_2_ titration from 0.21 to 1.0, a humidifier, heated inspiratory circuits, and a wide-bore nasal prong. The application of HFNO at a flow rate of 2 L/kg/min in small infants is reported to generate a splinting pressure of 4 to 6 cmH_2_O and helps maintain the upper airway in children under sedation [7]. Additionally, the anatomical nasopharyngeal dead space is rapidly washed out and prevents carbon dioxide retention. The applied positive pressure prevents micro-atelectasis, increases end-expiratory lung volume, and improves the effectiveness of ventilation and oxygenation in patients undergoing sedation. Further advantages are that F_I_O_2_ can be accurately adjusted between 0.21 and 1.0, and that iNO can be administered with ease. These are particularly effective in the setting of diagnostic cardiac catheterization where acute vasoactivity testing can be easily performed, and iNO can be swiftly initiated in case of abrupt elevation of PVR. The inability to monitor end-tidal capnography may be a potential disadvantage, although this can be managed by transcutaneous carbon dioxide monitoring or close respiratory monitoring along with intermittent blood gas sampling. The recommended flow rate for children weighing 16 to 30 kg is up to 35 L/min as was applied for this case.

This case report demonstrates the effectiveness of the combination of ketamine-dexmedetomidine sedation and HFNO in pediatric patients with severe pulmonary arterial hypertension and contributes to enhancing the safety of procedural sedation in this most vulnerable patient group. The presented strategy may also benefit younger children as ketamine-dexmedetomidine combination has been proved to be safe and effective for procedural sedation in pediatric patients including infants, and HFNO has been reported to be effective in preventing upper airway obstruction during procedural sedation in young children [8–13].

## Data Availability

Not applicable.

## Abbreviations

PH: Pulmonary hypertension
PAH: Pulmonary arterial hypertension
HFNO: High-flow nasal oxygen
PAP: Pulmonary arterial pressure
PVR: Pulmonary vascular resistance
SVR: Systemic vascular resistance
iNO: Inhaled nitric oxide
F_I_O_2_: Fraction of inspired oxygen
